# Weibull parametric model for survival analysis in women with endometrial cancer using clinical and T2-weighted MRI radiomic features

**DOI:** 10.1186/s12874-024-02234-1

**Published:** 2024-05-09

**Authors:** Xingfeng Li, Diana Marcus, James Russell, Eric O. Aboagye, Laura Burney Ellis, Alexander Sheeka, Won-Ho Edward Park, Nishat Bharwani, Sadaf Ghaem-Maghami, Andrea G. Rockall

**Affiliations:** 1 Department of Surgery and Cancer, Imperial College Hammersmith Campus, Du Cane Road, London, W12 0NN UK; 2 Chelsea and Westminster Hospital, 369 Fulham Rd, London, SW10 9NH UK; 3 The Imaging Department, Imperial College Healthcare NHS Trust, Hammersmith Hospital, Du Cane Road, London, W12 0HS UK; 4 Imperial College Healthcare NHS Trust, Hammersmith Hospital, Du Cane Road, London, W12 0HS UK

**Keywords:** Weibull parametric survival model, Survival analysis, Radiomics, Endometrial cancer, MRI, Cox proportional hazards model

## Abstract

**Background:**

Semiparametric survival analysis such as the Cox proportional hazards (CPH) regression model is commonly employed in endometrial cancer (EC) study. Although this method does not need to know the baseline hazard function, it cannot estimate event time ratio (ETR) which measures relative increase or decrease in survival time. To estimate ETR, the Weibull parametric model needs to be applied. The objective of this study is to develop and evaluate the Weibull parametric model for EC patients’ survival analysis.

**Methods:**

Training (*n* = 411) and testing (*n* = 80) datasets from EC patients were retrospectively collected to investigate this problem. To determine the optimal CPH model from the training dataset, a bi-level model selection with minimax concave penalty was applied to select clinical and radiomic features which were obtained from T2-weighted MRI images. After the CPH model was built, model diagnostic was carried out to evaluate the proportional hazard assumption with Schoenfeld test. Survival data were fitted into a Weibull model and hazard ratio (HR) and ETR were calculated from the model. Brier score and time-dependent area under the receiver operating characteristic curve (AUC) were compared between CPH and Weibull models. Goodness of the fit was measured with Kolmogorov-Smirnov (KS) statistic.

**Results:**

Although the proportional hazard assumption holds for fitting EC survival data, the linearity of the model assumption is suspicious as there are trends in the age and cancer grade predictors. The result also showed that there was a significant relation between the EC survival data and the Weibull distribution. Finally, it showed that Weibull model has a larger AUC value than CPH model in general, and it also has smaller Brier score value for EC survival prediction using both training and testing datasets, suggesting that it is more accurate to use the Weibull model for EC survival analysis.

**Conclusions:**

The Weibull parametric model for EC survival analysis allows simultaneous characterization of the treatment effect in terms of the hazard ratio and the event time ratio (ETR), which is likely to be better understood. This method can be extended to study progression free survival and disease specific survival.

**Trial registration:**

ClinicalTrials.gov NCT03543215, https://clinicaltrials.gov/, date of registration: 30th June 2017.

**Supplementary Information:**

The online version contains supplementary material available at 10.1186/s12874-024-02234-1.

## Background

Endometrial cancer (EC) is the sixth most common cancer in women with 417 000 new diagnoses made globally in 2020 [1]. To stratify patient risk for treatment planning, it is important to study the time between the diagnosis of EC and events such as death or recurrence which are of clinical interest. Survival analysis methods such as nonparametric Kaplan–Meier (KM) method and semi-parametric Cox proportional hazards (CPH) regression models have been proposed to study time to recurrence and death following the diagnosis of EC [2–6]. For the semi-parametric CPH method, different covariates have been included in the regression model, from social economic factors [7] to clinical and radiomic factors [6].

However, there are limitations in applying these nonparametric and semiparametric methods. Firstly, non-parametric methods such as KM estimate cannot be used for multivariate analysis as it can only be applied to study the effect of one factor at one time [2] and it also cannot handle the time varying covariate [8]. Secondly, although the semiparametric CPH method [3, 9] offers much greater flexibility than most parametric approaches, because it does not need to know the baseline hazard function, it cannot estimate event time ratio (ETR) which measures relative increase or decrease in survival time of EC survival data. Finally, the CPH method assumes that the continuous predictors are in linear association with log-hazard [3]. However, this assumption may not be true in some real clinical situations for EC data.

Nevertheless, a fully parametric model, if it is the appropriate parametric model, does offer many advantages [10, 11]. Indeed, the originator of the CPH model has expressed a preference for parametric modelling [12]. Because fully-specified models can be more convenient for representing complex data structures, and it can help with out-of-sample prediction [13]. Furthermore, a parametric model provides somewhat greater efficiency since fewer parameters are required to be estimated. Finally, it is easier to interpret the results if the parametric model matches some underlying mechanism associated with the data. In spite of these advantages, to the best of our knowledge, parametric models and the advantage of this method have not been investigated for EC survival analysis with radiomic features. The purpose of this study is to investigate and validate the Weibull parametric model for EC survival analysis based on clinical and radiomic features.

## Methods

This retrospective study protocol was approved by the institutional review board (IRB), and the research ethics committee of Imperial College Research Ethics Committee (ICREC) study reference number is 17/LO/0173 [6, 14]. The requirement for written informed consent was waived by the ethics committee (ICREC) because of the retrospective nature of the study. All experiments were conducted in accordance with the Declaration of Helsinki. This retrospective study will develop and test a model which will be further validated as part of a larger prospective study (ClinicalTrials.gov NCT03543215, https://clinicaltrials.gov/, date of the trial registration was 30th June 2017). In addition to three subjects were collected in the years of 2007, 2008 and 2010, the clinical data and image data were collected in between February 2012 and December 2021, and the patient’s information such as death were updated on 23rd May 2023.

### Patient information

**Fig. 1:**
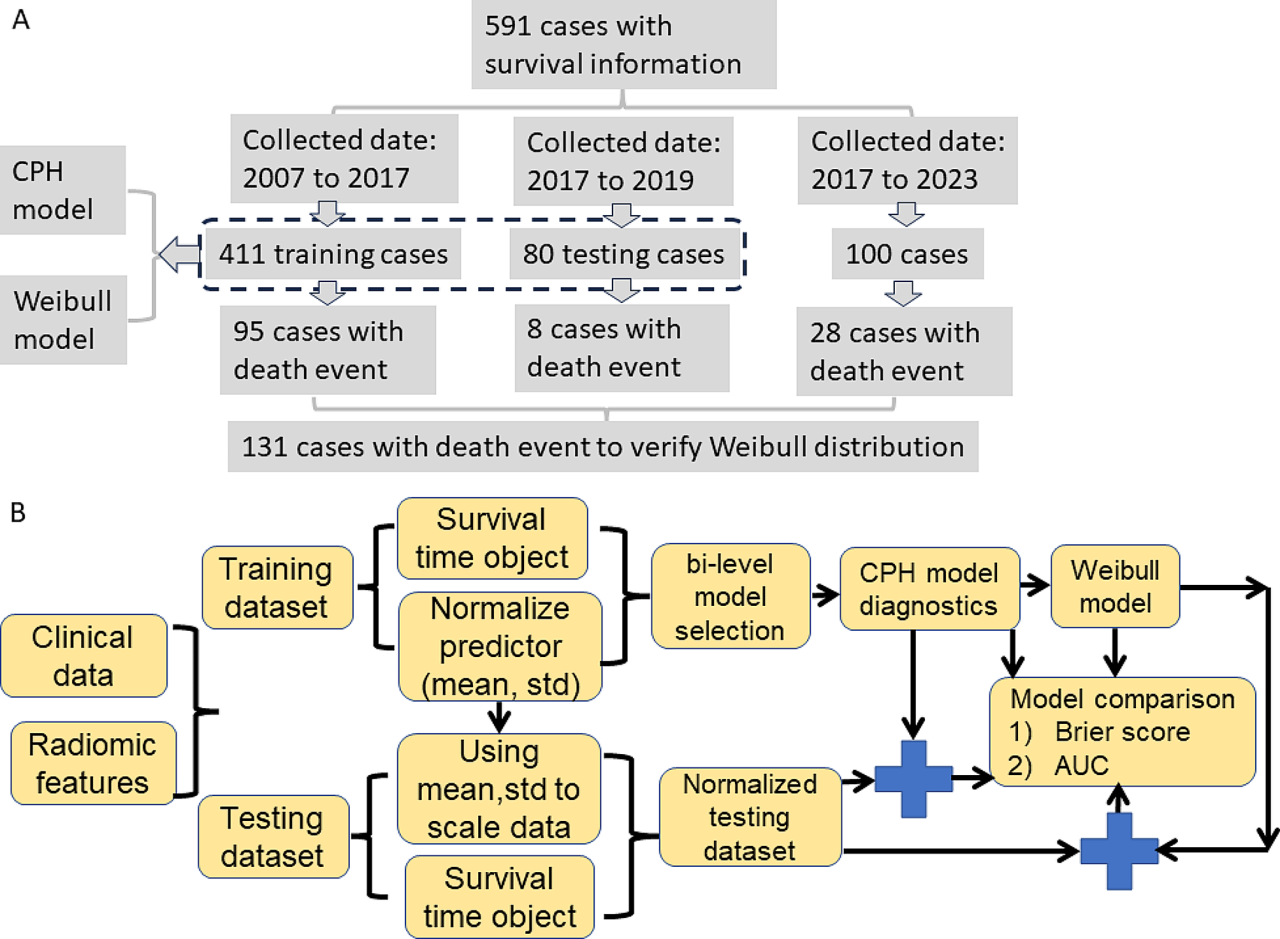
**A**, consort diagram for this study; **B**, Data analysis pipeline. std: standard deviation; CPH: Cox proportional hazard

Initially, 591 patients were included in the study. A consort diagram for this study is displayed in Fig. 1A. The inclusion and exclusion criteria for the dataset were: (1) availability of censoring or event (death) survival information, (2) availability of age at diagnosis and surgery date, (3) no other type of co-existing cancer [6]. The demographics of the dataset are available in Table 1. To investigate the survival probability, 131 patients with death time were selected to study the suitableness of the Weibull parametric method (Fig. 1A; Table 1). The missing values were neglected for group comparison in Table 1. Four hundred and ninety-one subjects with T2-weighted MRI scans were identified for clinical and radiomics integrated model development, of which 411 subjects were included as training dataset, and 80 subjects were used as testing dataset (Table 1). The radiomic features from MRI were obtained from a previous study [6].

### Weibull parametric model theory

From the well-known accelerated failure time (AFT) model we have [9]:

1 $$Y=\text{log}\left(T\right)=\mu +{\alpha }^{{\prime }}Z+\sigma \epsilon$$

where T is the survival time, µ is intercept, $Z$ is a $n\times p$ real matrix, n is the number of samples/subjects and p is the number of predictor/covariate; ${\alpha }^{{\prime }}$is the coefficient of the predictor. $\epsilon$ is a random error term assumed to follow the extreme value distribution. For Weibull distribution there is an additional parameter σ, which scales $\epsilon$. Let

2 $$\gamma =\frac{1}{\sigma }$$

3 $$\lambda ={e}^{-\frac{\mu }{\sigma }}$$

4 $$\beta =-\frac{\alpha }{\sigma }$$

Then we have a Weibull model with baseline hazard of [15]:

5 $$h\left(x|z\right)=\left(\gamma \lambda {t}^{\gamma -1}\right){e}^{{\beta }^{{\prime }}Z}$$

where γ is the shape parameter, and λ is the scale parameter. The hazard ratio (HR) is defined as:

6 $$HR={e}^{{\beta }^{{\prime }}}$$

Based on Weibull model (e.g. Eq. (5)), it is possible to estimate the event time ratio (ETR) [16, 17] which quantifies the relative difference in time it takes to achieve the *p*th percentile (95% in this study) between two levels of a covariate. The *p*th percentile of the (covariate-adjusted) Weibull distribution occurs at:

7 $${t}_{p}={\left[\frac{-\text{l}\text{o}\text{g}\left(p\right)}{\lambda {e}^{{\beta }^{{\prime }}Z}}\right]}^{1/\gamma }$$

Then the ratio of times for a covariate with value ${z}_{1}$ versus values ${z}_{0}$, with parameter estimate $\beta$ can be calculated as [17]:

8 $$\frac{{t}_{A}}{{t}_{B}}={\left[\frac{-\text{l}\text{o}\text{g}\left(p\right)}{\lambda {e}^{{\beta }^{{\prime }}{z}_{1}}}\right]}^{1/\gamma }/{\left[\frac{-\text{l}\text{o}\text{g}\left(p\right)}{\lambda {e}^{{\beta }^{{\prime }}{z}_{0}}}\right]}^{1/\gamma }={e}^{\frac{\beta ({z}_{0}-{z}_{1})}{\gamma }}$$

The Weibull model is unique in that it is simultaneously both proportional and accelerated so that both relative event rates and relative extension in survival time can be estimated, the latter being of clear clinical relevance. In Weibull model, as event time and event rate ratios are therefore linked by the shape parameter, it follows that if the HR can be estimated in a Weibull analysis, then so can the ETR be calculated by:

9 $$ETR={e}^{-\beta /\gamma }$$

For each covariate, $ETR={e}^{-{\beta }_{i}/\gamma }$, where ${\beta }_{i}$ is the coefficient of $i$ covariate. ETR is to qualify treatment effect is of some clinical relevance and is likely to be better understood. It is also known as “acceleration factor” which measures relative increase or decrease in survival time.

### Datasets and appropriateness for Weibull distribution

The distribution of the data was investigated based on 131 cases with death information from all 591 subjects (Fig. 1A; Table 1), and a two-parameter (shape and scale) Weibull model was proposed to fit the data. The appropriateness of using Weibull, lognormal, and log-logistic distributions for EC survival data were studied with Kolmogorov-Smirnov (KS) statistic test, quantile-quantile (Q-Q) plot, probability-probability (P-P) plot, and cumulative distribution function. A Q–Q plot is a plot of the quantiles of two distributions against each other, or a plot based on estimates of the quantiles. The pattern of points in the plot is used to compare the two distributions. A P–P plot can be used as a graphical adjunct to a test of the fit of probability distributions, with additional lines being included on the plot to indicate either specific acceptance regions or the range of expected departure from the 1:1 line.

**Table 1:** Clinical information from the subjects

			Training (*n* = 411)	Testing (*n* = 80)	*P* value (training testing difference)	Death (*n* = 131) for testing Weibull distribution
Age at diagnosis			66.72 ± 11.43	63.66 ± 12.16	***0.04***	70.18 ± 10.86
Grade					0.2231	(*n* = 127), 4 missing
		**1**	123	42		12
		**2**	130	19		14
		**3**	158	19		101
Risk score					0.2381	(*n* = 122), 9 missing
	**Low**		149	41		12
	**Intermediate**		78	15		10
	**High**		95	9		42
	**Advanced**		89	15		58

### Statistical analysis

The R software (R Foundation for Statistical Computing, Vienna, Austria; version 4.2.2 [18]), was used for statistical analysis. Particularly, cv.grpsurv() function from model selection package “grpreg” (version 3.4.0 [19]), was applied to determine the covariates in the CPH model. In the method, the maximum iteration was 1 million, and the composite minimax concave penalty (cMCP) was adopted for model selection. Other R packages, including “survival” (version 3.4-0 [20]), “survminer” (version 0.4.9 [21]), “eha” (version 2.10.1 [22]), “flexsurv” ( version 2.2.1 [23]), “SurvRegCensCov” (Version 1.5 [24]), and “fitdistrplus” packages (version 1.1.8 [25]) were used to fit the data into CPH model and Weibull models. The “rms” package (version 6.6-0 [26]) was applied for parametric survival model, and “riskRegression” package (version 2023.03.22 [27]) was employed to calculate the time-dependent area under the receiver operating characteristic curve (AUC) and Brier score. To generate time dependent AUC and Brier curves, the Score() function in “riskRegression” package was employed, and the bootstrap number was set to be 10 in the function. As the bootstrap shows randomness, so is the result.

### Data analysis pipeline

The clinical data and radiomics features were processed according to Fig. 1B, which shows the pipeline for fitting the data to a Weibull parametric model. Overall survival was studied, i.e., the dependent variable was the event (death) and time (time from diagnosis to death or time to the end of the study if the subject was still alive). Clinical data including patient’s age at diagnosis, clinical cancer grade, and risk score were collected from all 491 cases. After image processing, 2083 radiomic features have been extracted in the same way as previous studies [6, 14]. Both clinical and 2083 radiomic features were  included for the model selection [6]. Before applying model selection, the mean and standard deviation (std) of the training dataset were calculated for each numerical feature for Z-score normalization and then applied to normalize the testing dataset. Within the framework of CPH model, a bi-level model section method with a composite minimax concave penalty was adopted to select the final features from the training dataset for the survival analysis [28]. To validate the CPH model, diagnostic analysis was carried out using Schoenfeld test and the cox.zph() function from R “Survival” libraries [20]. The cox.zph() function is designed to test the proportional hazards assumption for a Cox regression model fit. The null hypothesis of the test is that the coefficient for the predictor does not vary with time. Residuals for each covariate in the CPH model were plotted to check the linearity of each covariate in the model.

For the AFT Weibull parametric model (e.g. Eq. (5)), the same selected features from CPH model were also used for the model fitting. The goodness of the Weibull parametric model fitting was evaluated with training and testing datasets. In addition, the check.dist() function from the R “eha” package was applied to check for the Weibull model fitting [22].

## Results

The CPH model selection results from the bi-level method are included in the supplementary information (Figure S1). Briefly, based on the training dataset, we obtained 5 features in the CPH model, i.e., age at diagnosis ($Age$), cancer grade ($Grade$), and three radiomic features, i.e., Gray level Difference Method (GLDM, original_gldm_LargeDependenceHighGrayLevelEmphasis), Gray Level Size Zone (GLSZM, GLSZM_GlVarianc_HLH_32gl), and Gray Level Run Length Matrix feature (GLRLM, GLRLM_LRLGLE_LHL_4gl). Therefore, the final semi-parametric CPH model is:

10 $$\eqalign{\text{log}\left(\frac{h\left(t\right)}{{h}_{0}\left(t\right)}\right) & = {a}_{1}Age+{a}_{2}Grade+{a}_{3}GLDM \cr & +{a}_{4}GLSZM+{a}_{5}GLRLM }$$

where $h\left(t\right)$is the hazard function determined by these covariates, ${h}_{0}\left(t\right)$ is called the baseline hazard, $Age,Grade,GLDM,GLSZM,\text{a}\text{n}\text{d} GLRLM$ are predictors; ${a}_{1},{a}_{2},{a}_{3},{a}_{4}$, and ${a}_{5}$ are the associate coefficients. As the dataset is not very big, and the model has not been evaluated on additional external datasets, the model selection process should not be taken as final. From the training dataset (*n* = 411), the mean and standard deviation of these numerical covariates can be found in Table S1.

Using the training dataset (411 cases), overall survival time was fitted into the CPH model as shown in Eq. (10). Because the CPH model was based on proportional hazard assumption, a statistical Schoenfeld test method was used to verify the assumption [29]. We did not find statistical significance (at *p* = 0.05) for each predictor based on cox.zph(), with the smallest *p* value of 0.055 for GLRLM variable in the CPH model, suggesting the proportional hazard assumption does hold in this analysis. In addition, we plotted the scaled Schoenfeld residual of each covariate for the Cox model fitting (Figure S2 and Table S2 in supplementary materials).

The suitableness to use Weibull model was verified using 131 EC subjects with death information (Figure S3 in the supplementary materials). From Figure S3, we concluded that the EC patient’s overall survival data is subject to the Weibull distribution, and we then applied the Weibull model for the analysis. To make it easier to compare with CPH model (Eq. (10)), we fit the data into the following Weibull function (Eq. (5)) which has the same predictors as the CPH model:

11 $$\eqalign {& h\left(x|z\right) \cr & =\left(\gamma \lambda {t}^{\gamma -1}\right){e}^{{a}_{1}Age+{a}_{2}Grade+{a}_{3}GLDM+{a}_{4}GLRLM+{a}_{5}GLSZM+\epsilon }}$$

where $\gamma$ is the shape parameter, $\lambda$ is the scale parameter, $t$ is time. ${a}_{1, }Age,{a}_{2},Grade,{a}_{3},GLDM,{a}_{4},GLRLM,{a}_{5},GLSZM$ have the same meanings as the CPH model (see Eq. (10)). Table 2 shows the estimated coefficients of the Weibull model (Eq. (11)) and CPH model (Eq. (10)) from training and testing datasets, respectively. From this equation, other parameters such as HR and ETR estimations (for Weibull model only) can be calculated.

Table 2 also shows the comparison results from Weibull and CPH models using training and testing datasets. For the training dataset, the coefficients between the Weibull and CPH models are similar except covariate cancer grade 2, which shows bigger differences between two models. This is also the case for the HR estimation results from two models. The maximum coefficient difference is from cancer grade 3, where the difference is less than 5%. Second, the ETR from the Weibull model is smaller than 1, suggesting that there is no increase in the overall survival interval for the patients, although the HR is larger than 1 signifying an increase of risk.

**Table 2:** Parameters estimation from Weibull and CPH models (confidence interval = 0.95) based on training and testing datasets. For the Weibull model with training dataset, the scale parameter lambda is 3.0447e-05, the shape parameter gamma is 0.9929. For the Weibull model with testing dataset, the scale parameter lambda is 1.2608e-07, the shape parameter gamma is 1.8057

Predictor (training data)	Weibull coefficients	Weibull HR	Weibull ETR	CPH coefficients	CPH HR
Age	0.5620	1.7541	0.5678	0.5476	1.7292
Grade 2	0.2038	1.2261	0.8144	0.2178	1.2433
Grade 3	1.9327	6.9080	0.1428	1.9048	6.7183
GLDM	0.2676	1.3068	0.7638	0.2738	1.3149
GLRLM	0.4012	1.4936	0.6676	0.3846	1.4691
GLSZM	0.1636	1.1778	0.8481	0.1546	1.1672
Predictor (Testing data)					
Age	0.2427	1.2746	0.8743	0.2123	1.2365
Grade 2	-0.6262	0.5346	1.4145	-0.7579	0.4687
Grade 3	0.8632	2.3707	0.6200	0.7667	2.1526
GLDM	1.3011	3.6733	0.4865	1.1876	3.2792
GLRLM	-0.6376	0.5286	1.4235	-0.5363	0.5849
GLSZM	-0.4999	0.6066	1.3189	-0.4383	0.6451

In addition, Table 2 includes the results from the testing dataset, comparing with training and testing datasets results, the coefficients and HR between the two models are bigger for these predictors. Additionally, from the testing dataset, three ETRs (cancer grade 2, GLRLM, and GLSZM covariates) from the Weibull model are bigger than 1, suggesting that there is an increase in the overall survival interval for the patients.

Clinically, there are 3 levels of cancer grade, i.e., grade 1–3. Because cancer grade is a categorical variable, only (n level − 1 = 2) level of cancer grades need to be included in the regression models (Eqs. 10 and 11).

**Fig. 2:**
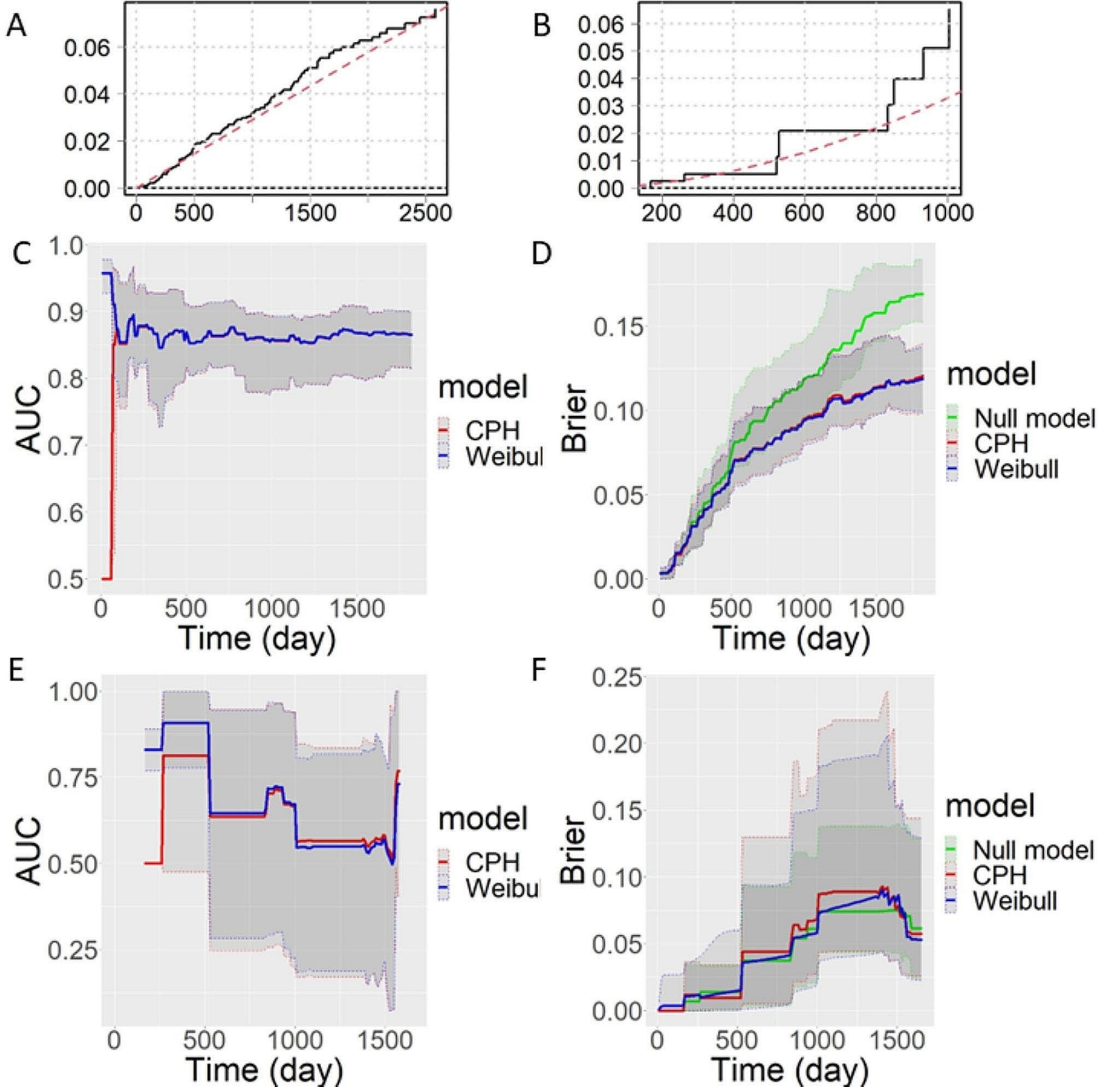
Graphical goodness of fit for Weibull model (**A, B**) and model comparison between the CPH and the Weibull models (**C, D, E, F**). A is generated from the training dataset, while B is from the testing dataset. The irregular black curves in A and B denote the nonparametric method, while the red smooth curve represents the Weibull model fitting. The unit of x-axis is day, which is the survival time. The closer between the red dotted line and the black curve, the better the fitting results. It compares the cumulative hazard function for non-parametric and parametric Weibull models. Time dependent AUC from training (**C**) and testing (**E**) datasets. Brier score from training data (**D**) and testing data (**F**) are also displayed

### Weibull model fitting diagnostic

The Weibull parametric model (Eq. (11)) fitting was diagnosed, i.e., the residual of the fitting was checked for the goodness of the fitting. Figure 2A and B display the graphical goodness of the fit results from training data (Fig. 2A) and testing data (Fig. 2B). The check.dist() function in R “eha” package was employed to generate Fig. 2A and B, and the y-axis in these figures is distance. Figure 2A and B compare the cumulative hazards functions for a non-parametric and a parametric model. The data fit the Weibull model well using the training dataset (Fig. 2A), but for the testing dataset (Fig. 2B), there is a bigger bias for the fitting after a time longer than 900 days. For each predictor in the model from training data (Fig. 2A; Table 2), except cancer grade 2, all other *p* values for the predictors are smaller than 0.05. For the whole Weibull model fitting, the overall model *p* value is 0 with degree of freedom of 6. If the Weibull model was fitted with testing data (Fig. 2B; Table 2), only *p* value from GLDM is smaller than 0.05, all the other predictors are larger than 0.05, but the overall model fitting *p*-value is 0.0013.

### Time dependent AUC and Brier score comparison between CPH and Weibull models

To evaluate the prediction accuracy, time dependent AUC (Fig. 2C and E) and Brier score (Fig. 2D and F) were calculated based on both training (Fig. 2C and D) and testing dataset (Fig. 2E and F). Figure 2C and E were generated for time dependent AUC estimation. Figure 2D and F were plotted for the Brier score curves, which include a null model (a model without predictor). The Brier score measures the prediction error, the smaller the value, the more accuracy of the model estimation. The mean and standard deviation of the AUC and Brier score from training and testing data can be found in Table S3.

For the AUC obtained from training dataset (Fig. 2C), the difference between CPH and Weibull model is mainly in the short time range survival time estimation. This is also the situation for testing data (Fig. 2E), where there is a larger difference between these two methods when the time range is smaller than 500 days, although the CPH model has larger AUC for the time range between 1000 and 1500 days.

## Discussion

This study applied and compared the use of the Weibull parametric model for patients with EC. We implemented the bi-level method to select the most important features for the CPH model, resulting in two clinical variables (age and cancer grade) and three radiomic features for inclusion in the regression model for survival analysis. To investigate whether the EC patient’s survival data is subject to Weibull distribution, 131 patients with death were included to fit into the Weibull parametric model and the results prove the suitability for the fitting (Figure S3). Based on the selected features from the CPH model, a Weibull model was fitted, and all the parameters including HR and ETR were computed based on training and testing datasets (Table 2). Diagnostic for the Weibull fitting and comparison with CPH model were conducted (Fig. 2A and B), the time dependent AUC and Brier results showed that it is better to use the Weibull parametric method for survival analysis (Fig. 2C ∼ Figure 2F).

There are several advantages to implementing the Weibull parametric model for EC patient survival analysis. Firstly, unlike the CPH model, where the baseline hazard function is unknown, in the parametric Weibull model the baseline hazard function can be estimated. Secondly, just like other parametric models, it can be used to predict the survival time even without having the samples in the training data, i.e., extrapolate the data out of the sample range. Once we obtain the model parameters, distribution function and probability density function can be computed. Thirdly, in addition to obtaining HR, we also get ETR from the model, which is beneficial to study the treatment effects on survival time. Finally, it makes the explanation of survival time stronger than the semi-parametric model like the CPH model. The Weibull method can interpret the survival time based on a specific distribution. As a result, the analysis with parametric models is stronger.

In summary, parametric Weibull model has the following advantages [16, 30]: firstly, Weibull analysis offers the opportunity to predict how data might mature over time, something that is of great interest within EC patient survival data. It can be an alternative method to CPH model to study not only overall survival, but progression free survival, and disease specific survival. Even when data do not follow an exact Weibull distribution, a Weibull-based analysis can give results that are very similar to those obtained from a Cox regression analysis.

It offers an easier way to interpret the results, and it can predict the survival probability out of sample. It also provides somewhat greater efficiency since fewer parameters are required to be estimated. Finally, Weibull analysis provides ETR values which are not available for semi-parametric models such as CPH model.

### Relation with other current studies

Although Weibull model has been applied to survival analysis for the patient with lung cancer study [31] and gastric cancer [32], to the best of our knowledge, this is the first time to apply Weibull parametric model with MRI radiomic features for survival analysis for the patients with EC. Using colon cancer patient data, a previous study [33] compared semi-parametric methods with parametric models based on Monte Carlo simulations study, even though semi-parametric models performed slightly better than the parametric approach, parametric models were superior to the semi-parametric model based on large dataset. However, another study shows the parametric model is generally better than the semi-parametric model for the survival study in cancer study [11]. Use of Weibull functions with overall survival significantly increases the precision of small arms typical of early phase trials. The study reported that frequent deviations from the CPH model proportional hazards assumption for the survival analysis due to treatment effect [11]. Furthermore, for cancer patients with treatments, parametric models have been applied and proved to be a plausible method [34], which is similar to our data where patients were recruited for treatment which may change the risk and, as a result, change the survival time.

### Limitations and further work

We have applied the Weibull parametric model for the study, other parametric models such as Gamma distribution, normal distribution [35], and modified Weibull models [36] have not been tested and compared for the study of EC survival. Therefore, one future direction is to apply and assess these models for EC survival analysis. Also, we did not evaluate the Weibull model for prediction of recurrent-event survival model for EC [37, 38]. The time to recurrence of EC patients may be estimated based on parametric recurrent event data analysis models. Furthermore, model selection for Weibull model [39] was not developed and used for this study. Instead, this study used the model selection algorithm for the CPH model, which may not be ideal for the Weibull model. Finally, although we included 491 cases in this study, the total number of patients with death is still small (*n* = 95 in training dataset). Larger dataset will be helpful to improve Weibull model fitting, as previous study showed that parametric model is superior to semi-parametric model when dataset is large for the estimation [33].

## Conclusion

We evaluated the Weibull parametric model for EC patient survival analysis. Our results demonstrate that the Weibull model is more accurate than a conventional CPH model containing the same number of features. The Weibull model calculates the treatment effect in terms of the HR and ETR simultaneously. ETR measures the relative improvement in survival time, and it is likely to be better understood by some non-statisticians than conventional HR. This method can be extended to study progression free survival and disease specific survival of EC patients.

### Electronic supplementary material

Below is the link to the electronic supplementary material.

Supplementary Material 1

## Data Availability

Data are unavailable due to privacy or ethical restrictions. Computer codes are available upon request.

## Abbreviations

AUC: Area under the receiver operating characteristic curve
CPH: Cox proportional hazard model
EC: Endometrial cancer
ETR: Event time ratio
FIGO: International Federation of Gynecology and Obstetrics
GLRLM: Gray Level Run Length Matrix
GLDM: Gray level Difference Method
GLSZM: Gray Level Size Zone
HR: Hazard ratio
KM: Kaplan–Meier

## References

[1] Crosbie EJ, Kitson SJ, McAlpine JN, Mukhopadhyay A, Powell ME, Singh N (2022). Endometrial cancer. Lancet.

[2] Kaplan EL, Meier P (1958). Nonparametric estimation from incomplete observations. J Am Stat Assoc.

[3] Cox DR (1972). Regression models and Life-Tables. J Roy Stat Soc: Ser B (Methodol).

[4] Clark TG, Bradburn MJ, Love SB, Altman DG (2003). Survival analysis part I: basic concepts and first analyses. Br J Cancer.

[5] Tejerizo-García Á, Jiménez-López JS, Muñoz-González JL, Bartolomé-Sotillos S, Marqueta-Marqués L, López-González G. Gómez JFP-R: overall survival and disease-free survival in endometrial cancer: prognostic factors in 276 patients. OncoTargets Therapy 2013:1305–13. 10.2147/OTT.S51532 PMC3787927 24092993

[6] Li X, Marcus D, Russell J, Aboagye EO, Ellis LB, Sheeka A, Park WE, Bharwani N, Ghaem-Maghami S, Rockall AG. An Integrated Clinical-MR Radiomics Model to Estimate Survival Time in patients with Endometrial Cancer. J Magn Reson Imaging 2022. 10.1002/jmri.28544 PMC10947322 36484309

[7] Bedir A, Abera SF, Vordermark D, Medenwald D (2022). Socioeconomic disparities in endometrial cancer survival in Germany: a survival analysis using population-based cancer registry data. J Cancer Res Clin Oncol.

[8] Snapinn SM, Jiang Q, Iglewicz B (2005). Illustrating the impact of a time-varying covariate with an Extended Kaplan-Meier Estimator. Am Stat.

[9] Bradburn MJ, Clark TG, Love SB, Altman DG (2003). Survival analysis part II: multivariate data analysis – an introduction to concepts and methods. Br J Cancer.

[10] Nardi A, Schemper M (2003). Comparing Cox and parametric models in clinical studies. Stat Med.

[11] Plana D, Fell G, Alexander BM, Palmer AC, Sorger PK (2022). Cancer patient survival can be parametrized to improve trial precision and reveal time-dependent therapeutic effects. Nat Commun.

[12] Reid N (1994). A conversation with Sir David Cox. Stat Sci.

[13] Aalen O, Borgan O, Gjessing H. Survival and event history analysis: a process point of view. Springer Science & Business Media; 2008.

[14] Li X, Dessi M, Marcus D, Russell J, Aboagye EO, Ellis LB, Sheeka A, Park W-HE, Bharwani N, Ghaem-Maghami S (2023). Prediction of deep myometrial infiltration, clinical risk category, histological type, and Lymphovascular Space Invasion in Women with Endometrial Cancer based on clinical and T2-Weighted MRI Radiomic features. Cancers.

[15] Klein JP, Moeschberger ML. Survival analysis: techniques for censored and truncated data. Volume 1230. Springer; 2003.

[16] Carroll KJ (2003). On the use and utility of the Weibull model in the analysis of survival data. Control Clin Trials.

[17] Haile SR. Weibull AFT Regression Functions in R. In. R help document; 2022.

[18] (2022) RCT: R: A language and environment for statistical computing. R Foundation for Statistical Computing, Vienna, Austria. URL https://www.R-project.org/. 2022.

[19] Breheny P, Huang J (2009). Penalized methods for bi-level variable selection. Stat its Interface.

[20] Therneau T. A package for survival analysis in R (R package version 3.5-0). In.: Springer: New York, NY, USA; 2023.

[21] Kassambara A, Kosinski M, Biecek P, Fabian S. survminer: Drawing Survival Curves using ‘ggplot2’. *R package version 03* 2017, 1.

[22] Broström G. Event History Analysis [R package eha version 2.10. 3]. 2023.

[23] Jackson CH. Flexsurv: a platform for parametric survival modeling in R. J Stat Softw 2016, 70. 10.18637/jss.v070.i08 PMC5868723 29593450

[24] Hubeaux S, Rufibach K. SurvRegCensCov: Weibull regression for a right-censored endpoint with a censored covariate. arXiv Preprint arXiv:14020432 2014.

[25] Delignette-Muller ML, Dutang C (2015). Fitdistrplus: an R package for fitting distributions. J Stat Softw.

[26] Harrell F Jr. rms: Regression Modeling Strategies. R package version 6.2-0. 2021. In.: Accessed 09/12/2021.) Available at: https://CRAN. R-project. org/package = rms; 2023.

[27] Gerds TA, Ozenne B. riskRegression: risk regression models and prediction scores for survival analysis with competing risks. *R package version* 2020, 5:2020.

[28] Breheny P, Huang J (2009). Penalized methods for bi-level variable selection. Stat Interface.

[29] Grambsch PM, Therneau TM (1994). Proportional hazards tests and Diagnostics based on weighted residuals. Biometrika.

[30] Haile SR. Weibull AFT Regression Functions in R. 2023.

[31] Ojara FW, Henrich A, Frances N, Nassar YM, Huisinga W, Hartung N, Geiger K, Holdenrieder S, Joerger M, Kloft C. A prognostic baseline blood biomarker and tumor growth kinetics integrated model in paclitaxel/platinum treated advanced non-small cell lung cancer patients. *CPT: Pharmacometrics & Systems Pharmacology*, n/a(n/a). 10.1002/psp4.12937 PMC10681433 36782356

[32] Esayas Lelisho M, Akessa GM, Kifle Demissie D, Fikadu Yermosa S, Andargie SA, Tareke SA, Pandey D. Application of Parametric Shared Frailty models to analyze Time-to-death of gastric Cancer patients. J Gastrointest Cancer 2022. 10.1007/s12029-021-00775-y 35064523

[33] Yenilmez İ, Yılmaz E, Kantar YM, Aydın D (2022). Comparison of parametric and semi-parametric models with randomly right-censored data by weighted estimators: two applications in colon cancer and hepatocellular carcinoma datasets. Stat Methods Med Res.

[34] Suh K, Carlson JJ, Xia F, Williamson T, Sullivan SD (2022). The potential long-term comparative effectiveness of larotrectinib vs standard of care for treatment of metastatic TRK fusion thyroid cancer, colorectal cancer, and soft tissue sarcoma. J Manag Care Spec Pharm.

[35] Wang P, Li Y, Reddy CK (2019). Machine Learning for Survival Analysis: a Survey. ACM Comput Surv.

[36] Lai CD, Min X, Murthy DNP (2003). A modified Weibull distribution. IEEE Trans Reliab.

[37] Ip EH, Efendi A, Molenberghs G, Bertoni AG (2015). Comparison of risks of cardiovascular events in the elderly using standard survival analysis and multiple-events and recurrent-events methods. BMC Med Res Methodol.

[38] Khan SA, Basharat N (2022). Accelerated failure time models for recurrent event data analysis and joint modeling. Comput Stat.

[39] Choi T, Choi S (2021). A fast algorithm for the accelerated failure time model with high-dimensional time-to-event data. J Stat Comput Simul.

