# Supplementary material for: Weibull parametric model for survival analysis in women with endometrial cancer using clinical and T2-weighted MRI radiomic features
Source: BMC Med Res Methodol. 2024 May 9;24:107. doi: 10.1186/s12874-024-02234-1 (PMC11080307; doi:10.1186/s12874-024-02234-1)
Supplement: Supplementary file 1 — Supplementary Material 1 [file 12874_2024_2234_MOESM1_ESM.docx]

**Supplementary materials**

Supplementary Methods 1: bi-level method for feature selection


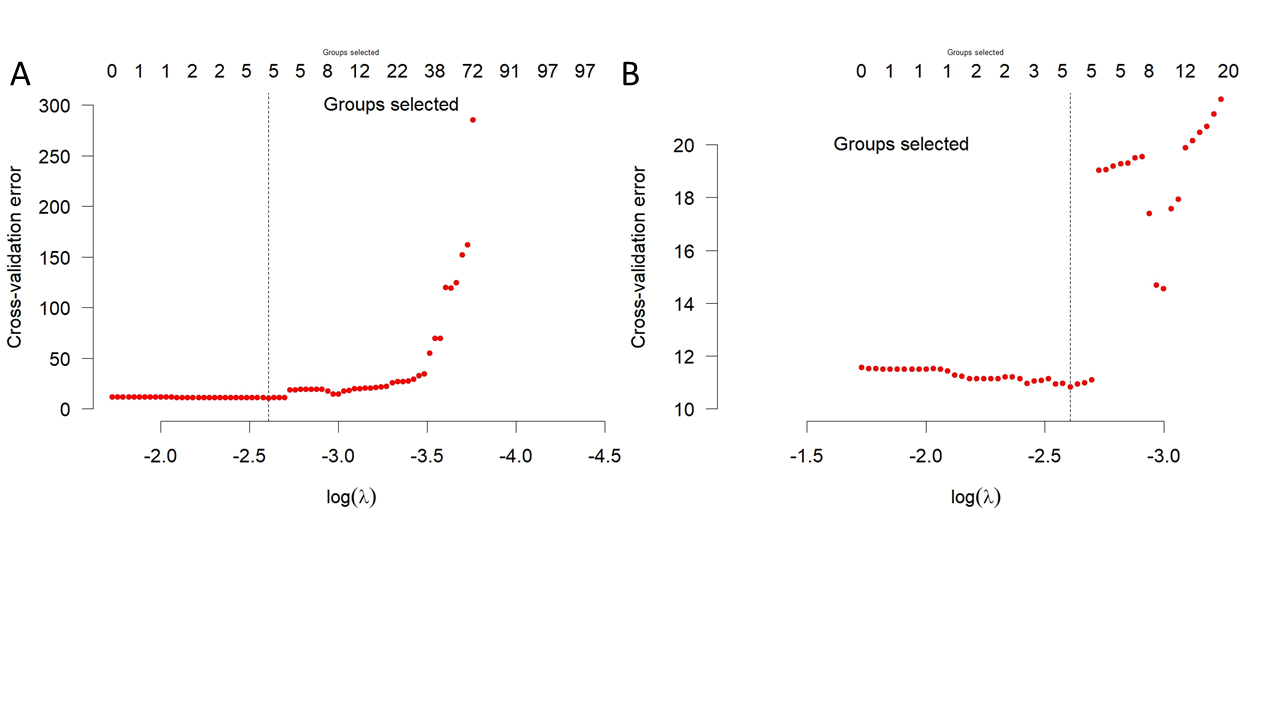


**Figure S1:** Model selection results. Y-axis is the cross-validation error; X-axis is the log value of the corresponding lambda/$\lambda$. The number on the top shows the corresponding number of covariates included. Figure S1 A shows the number of covariates change with $\lambda$; Figure S1 B is an amplified display of Figure S1 A around the minimum lambda ($\lambda$ =0.074).

Based on the training dataset, Figure S1 shows the Cox proportional hazards (CPH) model selection results from a bi-level model selection with composite minimax concave penalty. We can also see the impact of differences in $\lambda$ in the estimated coefficients. Figure S1 A shows the fitting cross-validation error (Y-axis) changes with $\lambda$ (X-axis). Figure S1 B is the zoomed version of Figure S1 A at the optimal (minimum) $\lambda$ value. As shown in Figure S1 B, when $\lambda$ is 0.074 (log(0.074)=-2.6), we have the smallest cross-validation error. We therefore selected the features included in the model when $\lambda$ =0.074. We obtained 5 features in the CPH model: age at diagnosis ($Age$); cancer grade ($Grade$)’ and three radiomic features: Gray level Difference Method (GLDM, original_gldm_LargeDependenceHighGrayLevelEmphasis); Gray Level Size Zone (GLSZM, GLSZM_GlVarianc_HLH_32gl); and Gray Level Run Length Matrix feature (GLRLM, GLRLM_LRLGLE_LHL_4gl). The mean and standard deviation of these numerical covariates can be found in Table S1. To use the model, features need to be normalized with Z-score method.

**Table S1. Mean and standard deviation (std) for each covariate in the train dataset.**

| Covariate | Age | Cancer grade | GLDM | GLSZM | GLRLM |
| --- | --- | --- | --- | --- | --- |
| Mean | 66.723 | Categorical variable (NA) | 2948.138 | 59.401 | 2.208 |
| Std | 11.426 | Categorical variable (NA) | 2037.828 | 36.136 | 4.434 |

Supplementary Methods 2: Schoenfield residuals

Figure S2 plots the scaled Schoenfeld residuals (circles) and their fitting results. From the plots, a visual check of the proportional hazard assumption was performed for each predictor. The curves in the figure are a spline function fit (by default, with 4 degrees of freedom) of the time varying estimates of the predictor. Proportionality is supported when the line is straight, and flat. The dashed lines are confidence intervals at two standard errors.

We have conducted the Schoenfeld proportional hazard test for all covariates in the selected model. The results showed non-significant (P<0.05) for non-proportional hazards (PH) covariates in the model (Table S2). If Schoenfeld test with p<0.05, then the feature meets the non-PH assumption. Both global and individual Schoenfeld test have p value larger than 0.05, suggesting the covariate meets the PH assumption.

**Table S2. Schoenfeld proportional hazard test (p value).**

| Covariate | Age | Cancer grade | GLDM | GLSZM | GLRLM | Global |
| --- | --- | --- | --- | --- | --- | --- |
| P value | 0.529 | 0.205 | 0.567 | 0.829 | 0.055 | 0.1925 |


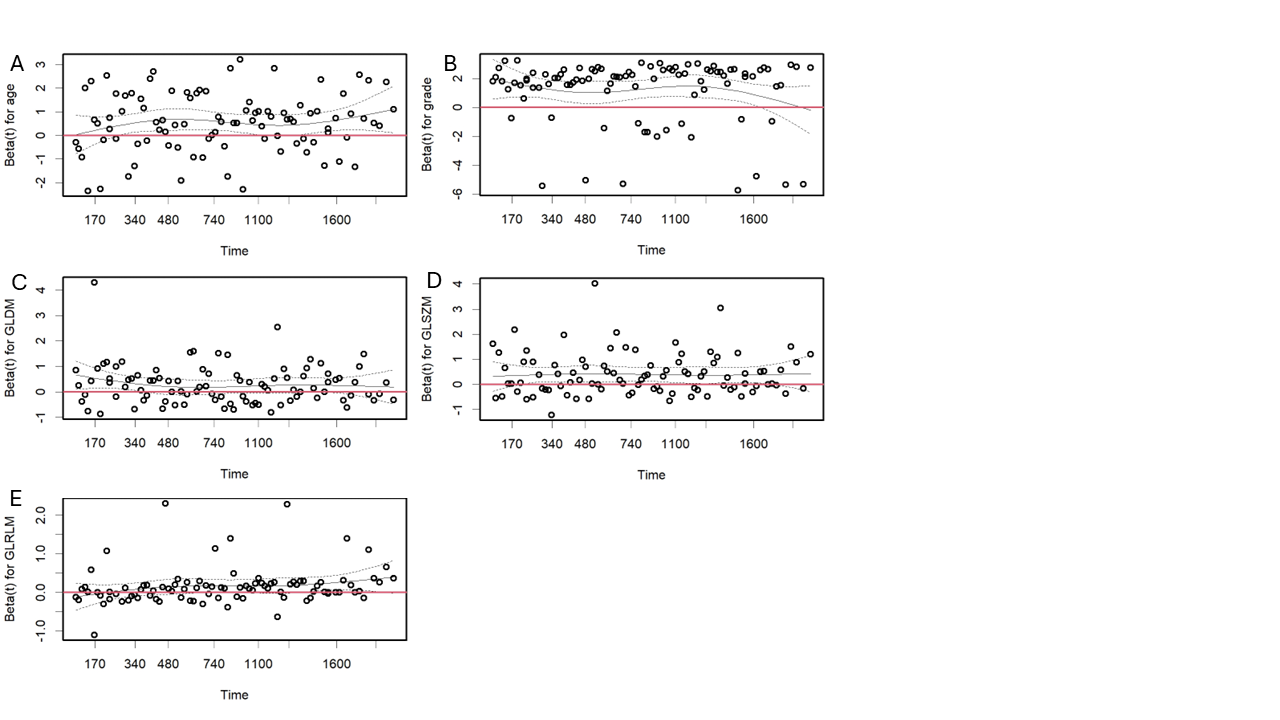


**Figure S2:** The scaled Schoenfeld residuals for each covariate: Cox proportional hazards model diagnosis, using the training dataset. The red line is the x-axis horizontal line. The unit of x-axis is day. Residuals are shown from the five selected features: age (A), cancer grade (B), GLDM (C), GLRLM (D), and GLSZM (E). The Schoenfeld proportional hazard test results are displayed in Table S2.

Supplementary Methods 3: Weibull model fitting

Using 131 endometrial cancer (EC) patients with death information, the appropriateness of fitting the data to a Weibull distribution was investigated. The survival time of these patients was fitted into a two-parameter Weibull model. Figure S3 A shows the results: the shape parameter was 1.4151, and the scale parameter was 940.3150. The Kolmogorov-Smirnov (KS) statistical test result was p=0.8436 (p value of the KS test) for the fitting. A high value in the KS test (maximum 1.0) indicates a good fit, whereas a low value (minimum = 0.0) indicates that the fit is not good. A KS value lower than 0.05 would suggest that the lack of fitting into a Weibull function is significant.

In addition, lognormal and log-logistic models were studied, and Figure S3 B displays the quantile-quantile (Q-Q) plots of Weibull, lognormal, and log-logistic parametric distributions. This plots the quantiles of two distributions against each other. The theoretical quantiles are plotted in the x-axis, against empirical quantiles in the y-axis. A 45-degree reference line is also plotted. The closer the data is to the 45-degree reference line, the better the good match between the theoretical quantile and empirical quantiles. This can be used to visually check the distribution of the data. Examining the Weibull distribution in comparison to both lognormal and log-logistic distributions visually, it is clear that the data is closer to the Weibull distribution.

Similarly, cumulative distribution function (CDF) for Weibull, lognormal, and log-logistic functions are displayed in Figure S3 C. In the figure, the x-axis includes the duration data (time, in days), while the y-axis is the value of CDF. Again, it is clear visually that the Weibull distribution is closer to the survival data than the lognormal and log-logistic functions. Figure S3 D displays a probability–probability (P-P) plot. Again, theoretical (x-axis) and empirical (y-axis) probabilities are plotted against each other. Similarly to the Q-Q plot, a 45-degree reference line is included to aid in visual inspection of the model; if the data generated from the theoretical distribution is closer to the reference line, it implies that the data is more suitable to fit that distribution. Although this is possibly more obvious on the Q-Q plot, the P-P plot confirms that the data fits a Weibull distribution slightly more closely than a lognormal or log-logistic distribution.


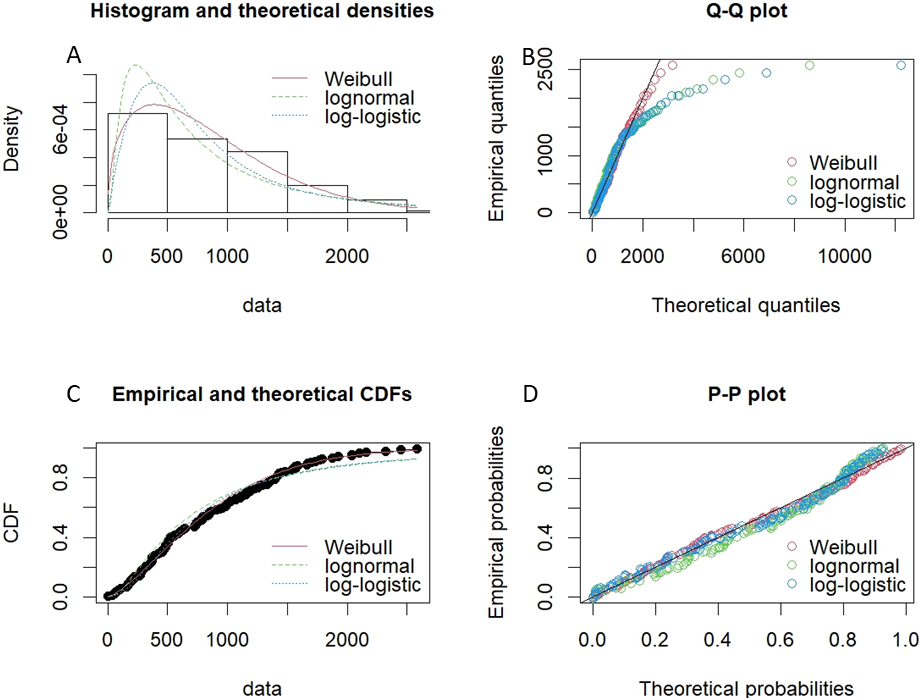


**Figure S3:** Weibull distribution fitting. Weibull parametric model for 131 EC death cases. A: Survival data histogram and Weibull, lognormal, log-logistic function fitting results. B: A quantile-quantile (Q-Q) plot for theoretical quantile (x-axis) and empirical quantile (y-axis). C: Cumulative distribution function (CDF) plot for Weibull, lognormal, and log-logistic functions. The dotted curve represents the data. D: A probability-probability (P–P) plot for theoretical probability (x-axis) and empirical probability (y-axis).

**Table S3. CPH model and Weibull model time dependent AUC and Brier score values from training and testing datasets.**

|  | Training  (AUC) | Training  (Brier) | Testing  (AUC) | Testing (Brier) | Concordance  Index |
| --- | --- | --- | --- | --- | --- |
| CPH (mean) | 0.8300 | 0.0808 | 0.6388 | 0.0494 | 0.8263 |
| CPH (Std) | 0.0626 | 0.0349 | 0.0992 | 0.0329 |  |
| Weibull (Mean) | 0.8451 | 0.0804 | 0.6754 | 0.0460 | 0.8269 |
| Weibull (Std) | 0.0237 | 0.0348 | 0.1358 | 0.0292 |  |

In Table S3, the mean AUC value from the Weibull model is larger than the AUC from the CPH model (Figure 2B and Figure 2C). This is consistent for testing data as shown, although the standard deviation of the AUC is larger than the Weibull model. For Brier score comparison (Figure 2D and 2F), Brier score from Weibull model was smaller for training dataset (Figure 2D) and testing dataset (Figure 2F), suggesting smaller model fitting error for the Weibull model. Both AUC and Brier score results indicate that the Weibull model is better than the CPH model for EC survival analysis. Finally, the concordance index from Weibull model is also slightly larger (see the last column of Table S3).
